# Morbidity Rate Prediction of Dengue Hemorrhagic Fever (DHF) Using the Support Vector Machine and the *Aedes aegypti* Infection Rate in Similar Climates and Geographical Areas

**DOI:** 10.1371/journal.pone.0125049

**Published:** 2015-05-11

**Authors:** Kraisak Kesorn, Phatsavee Ongruk, Jakkrawarn Chompoosri, Atchara Phumee, Usavadee Thavara, Apiwat Tawatsin, Padet Siriyasatien

**Affiliations:** 1 Computer Science and Information Technology Department, Faculty of Science, Naresuan University, Phitsanulok, Thailand; 2 National Institute of Health, Department of Medical Sciences, Ministry of Public Health, Nonthaburi, Thailand; 3 Department of Parasitology, Faculty of Medicine, Chulalongkorn University, Bangkok, Thailand; 4 Excellence Center for Emerging Infectious Disease, King Chulalongkorn Memorial Hospital, Thai Red Cross Society, Bangkok, Thailand; National Taiwan Ocean University, TAIWAN

## Abstract

**Background:**

In the past few decades, several researchers have proposed highly accurate prediction models that have typically relied on climate parameters. However, climate factors can be unreliable and can lower the effectiveness of prediction when they are applied in locations where climate factors do not differ significantly. The purpose of this study was to improve a dengue surveillance system in areas with similar climate by exploiting the infection rate in the *Aedes aegypti* mosquito and using the support vector machine (SVM) technique for forecasting the dengue morbidity rate.

**Methods and Findings:**

Areas with high incidence of dengue outbreaks in central Thailand were studied. The proposed framework consisted of the following three major parts: 1) data integration, 2) model construction, and 3) model evaluation. We discovered that the *Ae*. *aegypti* female and larvae mosquito infection rates were significantly positively associated with the morbidity rate. Thus, the increasing infection rate of female mosquitoes and larvae led to a higher number of dengue cases, and the prediction performance increased when those predictors were integrated into a predictive model. In this research, we applied the SVM with the radial basis function (RBF) kernel to forecast the high morbidity rate and take precautions to prevent the development of pervasive dengue epidemics. The experimental results showed that the introduced parameters significantly increased the prediction accuracy to 88.37% when used on the test set data, and these parameters led to the highest performance compared to state-of-the-art forecasting models.

**Conclusions:**

The infection rates of the *Ae*. *aegypti* female mosquitoes and larvae improved the morbidity rate forecasting efficiency better than the climate parameters used in classical frameworks. We demonstrated that the SVM-R-based model has high generalization performance and obtained the highest prediction performance compared to classical models as measured by the accuracy, sensitivity, specificity, and mean absolute error (MAE).

## Introduction

Dengue fever (DF) is one of the most important infectious diseases worldwide, particularly in areas with tropical and sub-tropical climates [1–3]. DF is a viral vector-borne disease and is spread by the female *Aedes aegypti* mosquito [4,5]. In Thailand, dengue hemorrhagic fever (DHF) was first reported in 1949 [6] and remains the country’s primary public health problem because no specific medical treatments [7] or effective vaccines [8] exist to prevent dengue infections. Therefore, several scientists have undertaken a massive endeavor to construct early warning systems using various predictors, statistical techniques, and mathematical models to facilitate early public health responses to reduce morbidity and mortality. Several factors are included in the models, including climate variables, mosquito density, human populations, demographic transitions, places (e.g., school, home, or hospital), and areas (e.g., towns and rural areas). Of these, climate or weather variables, particularly rainfall and temperature, are the most commonly used variables to increase the forecasting performance of predictive models [5,9–13]. Many works have indicated that higher rainfall results in a higher risk of dengue virus transmission [14–16]. However, some studies have revealed that rainfall is significantly negatively associated with dengue incidence because larvae and eggs can be washed away by massive rainfalls [17–19]. In addition, the climate-based model may be less efficient in areas that are geographically similar because these areas have only minor differences in temperature and rainfall. Therefore, relying only on the weather variables can lead to lower reliability of the model. Focks *et al*. [20,21] has constructed comprehensive models that take into account the majority factors known to influence dengue epidemiology and introduced to use a new factor, a number of *Ae*. *aegypti* paupae, for the prediction model. This study revealed that a number of *Ae*. *aegypti* paupae is more appropriate for assessing risk and directing control operations. Moreover, *Ae*. *aegypti* populations have been suggested for use in predicting dengue outbreaks [22,23]. Researchers have hypothesized that higher *Ae*. *aegypti* population densities result in higher dengue incidences. However, Goh et al. [24] reported that a high number of dengue cases were found in Singapore even though the *Ae*. *aegypti* populations decreased. This result indicates that *Ae*. *aegypti* populations are not positively related to the outbreaks. Therefore, we hypothesize that the infection rate of the dengue virus in *Ae*. *aegypti* is significantly correlated with the outbreaks and can be used to enhance the effectiveness of the morbidity rate prediction. Moreover, choosing the forecasting technique is an important task for this study.

Many classical forecasting techniques, such as the autoregressive integrated moving average (ARIMA) [13], the K-H model [25], the seasonal autoregressive integrated moving average (SARIMA) [5,12,26], the SVM [27], a Poisson regression analysis [28], and the artificial neural network (ANN) have been used to extrapolate dengue incidences [29]. Time series analysis methodologies, particularly ARIMA and SARIMA [26], have been increasingly deployed in the field of epidemiological research on DF, DHF, and many other infectious diseases. Typically, a time series analysis technique involves homogeneous data over long periods. The main drawback of this method is that it does not consider other variables that are associated with the dependent parameter. Nonetheless, many research studies have revealed that the severity of dengue outbreaks is influenced by several independent variables, and particularly by climate factors. Therefore, a time series analysis may be less reliable because it only considers the historical data of the dependent variable. The ANN has been widely used in biomedical fields for data analysis and modeling, forecasting, and diagnostic classification. For dengue outbreaks, the ANN has been used for noninvasive diagnosis of the risk in dengue patients [8, 23] by exploiting four parameters (day of fever, reactance, gender, and the risk group quantification) to classify patients into risk groups. The overall prediction accuracy to perform such a task was 96.86%. Dengue outbreak prediction in Malaysia using the ANN was proposed by Husin *et al* [30]. The experiments showed that the ANN-based model outperformed a nonlinear regression model, as measured by the mean square error (MSE). However, the ANN needs to determine how many neurons are required for a task, and this is another issue that affects whether the ANN is optimized. A new machine learning method, e.g., the SVM, has become more attractive to researchers in the field. SVM is an efficient approach for nonlinear classification problem solving. Benjamin *et al*. [31] reported using the SVM to predict dengue incidence in Singapore and Bangkok using a search query from Google and demonstrated that SVM models outperformed logistic regressions for predicting high periods of dengue incidence. Similar to the work of Shameem *et al*. [27], the SVM displayed a more accurate predictive performance for dengue cases. The SVM is an efficient technique based on statistical learning theory for nonlinear classification problem solving and has been reported to be efficient in generalization performance; the SVM has been implemented in various fields [32]. The SVM can solve several problems of classification techniques such as the overfitting problem and the curse of dimensionality. The SVM has been proven to provide more solutions to boundaries compared to the neural network (NN) and can solve local minimum problems found in the NN [33].

An effective prediction model of dengue outbreaks is of great importance to relevant public health decision makers who are typically responsible for budgets and manpower. Hence, this paper aimed to use the SVM to construct a predictive model for dengue incidence using climate parameters and the newly discovered variable, the *Ae*. *aegypti* mosquito infection rate, which has never been used in any existing predictive models.

## Materials and Methods

### Study areas

Three provinces (Nakhon Pathom, Ratchaburi, and Samut Sakhon) in the central region of Thailand were selected for this experiment. In each province, data regarding *Ae*. *aegypti* larvae and adults were collected from 2007 to 2013. Nakhon Pathom is located at 13.9167°N, 100.1167°E and covers an area of 2,168.327 km^2^. The population size is 882,184, and the population density is 406.85 people/km^2^. Ratchaburi is located at 13.5289°N, 99.8144°E, and covers an area of 5,196.462 km^2^ with a population of 850,162 and a population density of 163.60 people/km^2^. Samut Sakhon is located at 13.5472°N, 100.2736°E. The population size of Samut Sakhon is 519,457 with an area of 872.347 km^2^, corresponding to a high population density of 595.47 people/km^2^. These provinces were selected for the following three major reasons: 1) a high DHF morbidity rate as reported in Thailand health information system (www.hiso.in.th), 2) a high mosquito density, and 3) a minor difference in climatic factors. Human baiting was performed by officers with highly experience from National Institute of Health, Thailand and the study was approved by the Ethic Committee of Research Affairs Unit, Faculty of Medicine, Chulalongkorn University (COA No. 328/2014).

### Key predictors for the proposed model

Due to the high cost of collecting mosquito infection data, seasonal data collection was performed at this stage of the experiment. Here, nine major contributors to dengue epidemics, collected between 2007 and 2013, were included in the model. These are temperature, rainfall, humidity, wind speed, *Ae*. *aegypti* larvae infection rate, male mosquito infection rate, female mosquito infection rate, population density, and morbidity rate. All data required cleaning before being entered into a model. This process included data transformation and the removal of missing values. The values of the dengue virus infection rate in mosquitoes were obtained from a previous report by Chompoosri *et al* [7]. The *Ae*. *aegypti* larvae were collected from indoor clean water containers, and the adult mosquitoes were collected using human bait twice a season between 2007 and 2012. The collected larvae and mosquitoes were visually identified for *Ae*. *aegypti* species. Dengue virus was detected by pooling and storing all *Ae*. *aegypti* larvae and mosquitoes in cryogenic vials (5 larvae or mosquitoes/pool/vial) in liquid nitrogen.

### Predictive model construction based on SVM

The SVM was first introduced in 1992 by Boser, Guyon, and Vapnik in COLT-92 [34] and received much attention from researchers due to its tendency to have better empirical performance. The SVM is a prediction tool that uses machine-learning theory to maximize predictive accuracy while automatically avoiding overfitting of the data. Three major components [35] of the SVM include learning theory [36], the optimal hyperplane algorithm [35], and the kernel functions.

Several kernel functions are used with the SVM. For instance, the linear kernel is the simplest kernel function (Eq 1).

k(x,y)=x⋅y+c (1)

The polynomial kernel (Eq 2) is a classical method for non-linear modeling and is well fitted for problems when all the training data are normalized.

$$\text{k}(\text{x},\text{y})={\left(\text{x}\cdot \text{y}\right)}^{\text{d}}$$ (2)

The radial basis function (RBF) kernel is one of the most powerful kernels (Eq 3).

$$\text{k}(\text{x},\text{y})=\text{exp}\left[-\frac{{∥\text{x-y}∥}^{2}}{{2\sigma }^{2}}\right]$$ (3)

These three kernels will be deployed in the dengue predictive model, and the highest accuracy model will be selected.

### Predictive model performance criteria

Sensitivity, specificity, and accuracy were used to assess the effectiveness of the proposed model. Sensitivity indicates the fraction of high-risk dengue outbreak areas that are correctly classified, whereas specificity shows the fraction low-risk areas that are correctly classified. These three measures are computed by the following equations:

$$\text{Sensitivity=}\frac{\text{TP}}{\text{TP+FN}}$$ (4)

$$\text{Specificity=}\frac{\text{TN}}{\text{TN+FP}}$$ (5)

$$\text{Accuracy=}\frac{\text{TP+TN}}{\text{TP+TN+FP+FN}}$$ (6)

where: True positive (TP) is the number of correctly classified high morbidity rates.

True negative (TN) is the number of correctly classified low morbidity rates.

False positive (FP) is the number of incorrectly classified high morbidity rates.

False negative (FN) is the number of incorrectly classified low morbidity rates.

Ideally, a perfect predictor would be described as 100% sensitive (i.e., predicting all areas from the high-risk group as being high risk) and 100% specific (i.e., not predicting any areas from the high-risk group being low risk).

### Definition 1: High morbidity rate of a dengue outbreak in Thailand

The morbidity rate of the dengue outbreak in Thailand is defined as the number of dengue cases per 100,000 population (Eq 7). The morbidity rate is high when it equals 50 or more; otherwise, the rate is classified as low.

$$\text{Morbidity rate=}\frac{\text{dengue case}\times 100,000}{\text{population density}}$$ (7)

### Methodology

**Fig 1:**
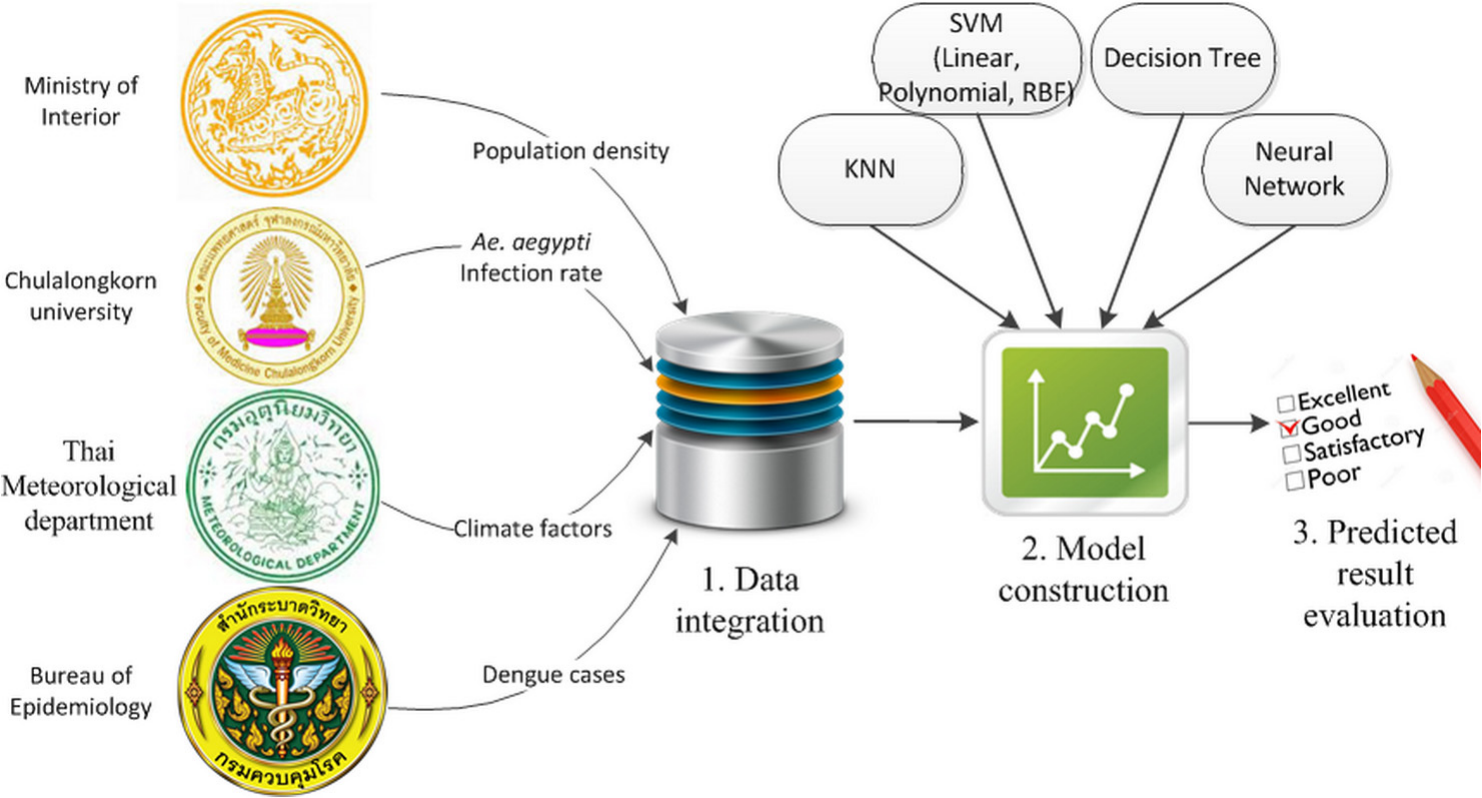
The experimental processes. The following three experimental processes are conducted: 1) data integration, 2) predictive model construction using Weka [37], and 3) result evaluation using sensitivity, specificity, and overall accuracy.

To develop a predictive model for dengue outbreaks, three main processes are defined as illustrated in Fig 1 and can be explained as follows:

1. ***Data integration***: All data from several sources, as shown in Table 1, were collected and integrated into a unified data storage system. Before feeding the collected data into a forecasting model, the data were extensively processed by data cleaning, transformation, categorization, and scaling. As a result, 648 samples remained.
2. ***Model construction***: Two well-known data mining techniques were applied for the prediction task, the K-nearest neighbor (*KNN*), the decision tree (DT), the NN, and the SVM with different kernels. These supervised classification algorithms were deployed in the models, and the predicted results were compared and analyzed. The SVM was selected because it has been proven to provide a better performance and processing time compared to the NN and has a good generalization performance [38].
3. ***Predicted result evaluation***: The predicted results will be compared to the observed data to assess the constructed model using all measures addressed in Eqs 4–6. In addition, a 10-fold cross-validation approach was exploited to measure the proposed model effectiveness. The cross-validation procedure can prevent the problem of overfitting. Because changing the parameters of the SVM directly affects the values of sensitivity, specificity, and overall accuracy, it is important to determine the optimal parameters to obtain the highest accuracy with a good sensitivity and specificity.

Table 1 lists the variables considered in the proposed forecasting model. The values of all variables were collected between 2007 and 2013

**Table 1:** Independent and dependent variables collected from various sources and used in the proposed forecasting model.

Variables	Sources	Unit
1. Temperature	Thai Meteorological Department	Celsius (C)
2. Rainfall	Thai Meteorological Department	Millimeters (mm)
3. Humidity	Thai Meteorological Department	Percentage (%)
4. Wind (speed)	Thai Meteorological Department	Miles per hour (mph)
*5*. *Ae*. *aegypti* larvae infection rate	Parasitology Department, Chulalongkorn University	Percentage (%)
6. Female mosquito infection rate	Parasitology Department, Chulalongkorn University	Percentage (%)
7. Male mosquito infection rate	Parasitology Department, Chulalongkorn University	Percentage (%)
8. Population density	Ministry of Interior	People
9. Dengue cases (*later used to compute morbidity rate*)	National Trustworthy and Competent Authority Epidemiological Surveillance and Investigation Department (NTCAESI)	Cases

Table 2 demonstrates more details of the three proposed processes in Fig 1. The data cleaning involved amending or removing data that were incorrect, incomplete, improperly formatted, or duplicated. These data should be removed from the model because they can lower the classification or forecasting power. Because data come from various sources, their format and structure are often different and not ready to be processed. Therefore, they need to be restructured into a format that the SVM tools can execute. In this experiment, all data were transformed into a CSV format. In addition, both techniques require that each data point is represented as a vector of a real number. Hence, a categorical variable, e.g., province and season, must be converted into a numerical value. Here, season was converted into *m* numbers to represent the *m*-season attribute. Only one of the *m* numbers is one, and others are zero. For example, a three-season attribute, such as summer, rainy, and winter, can be represented as (1,0,0), (0,1,0), and (0,0,1). This representation is more stable than using a single number, e.g., 1, 2, and 3, as suggested by Hsu *et al* [39].

**Table 2:** Experimental procedure.

1: Data cleaning: e.g., removing missing values
2: Formatting data to for SVM and Poisson regression, e.g., CSV
3: Categorizing the features of a model
4: Conducting simple scaling (standardization) of the features
5: Applying the forecasting techniques using:
1) K-Nearest Neighbor (*KNN*)
2) Decision Tree (DT)
3) Neural Network (NN)
4) Support Vector Machine using Linear kernel (SVM-L)
5) Support Vector Machine using Polynomial kernel (SVM-P)
6) Support Vector Machine using Radial basis function kernel (SVM-R)
6: Evaluating all models using these measures:
1) Sensitivity, specificity, and overall accuracy
2) 10-fold cross-validation
7: Selecting the best model

Data scaling refers to the method used to normalize the range of features or independent variables of a data mining model. This approach can ensure that data are not overwhelmed by each other in terms of distance measures [33]. We used a standardization technique (Eq 8) to rescale both the training and testing data:

$$\text{x}\prime =\frac{\text{x-mean}(f)}{\text{stddev}(f)}$$ (8)

where *x*′ is a standardized value, *x* is an original value, and *mean* and *stddev* are a median and a standard deviation value of each feature (*f*), respectively.

Having scaled the data, they are ready to be processed by the techniques mentioned in step 5 of Table 2; the results of each model will be assessed by sensitivity, specificity, and accuracy. Finally, the model that best optimizes those measures is selected.

## Results

### Mosquito infection rate and observed data analysis

**Fig 2:**
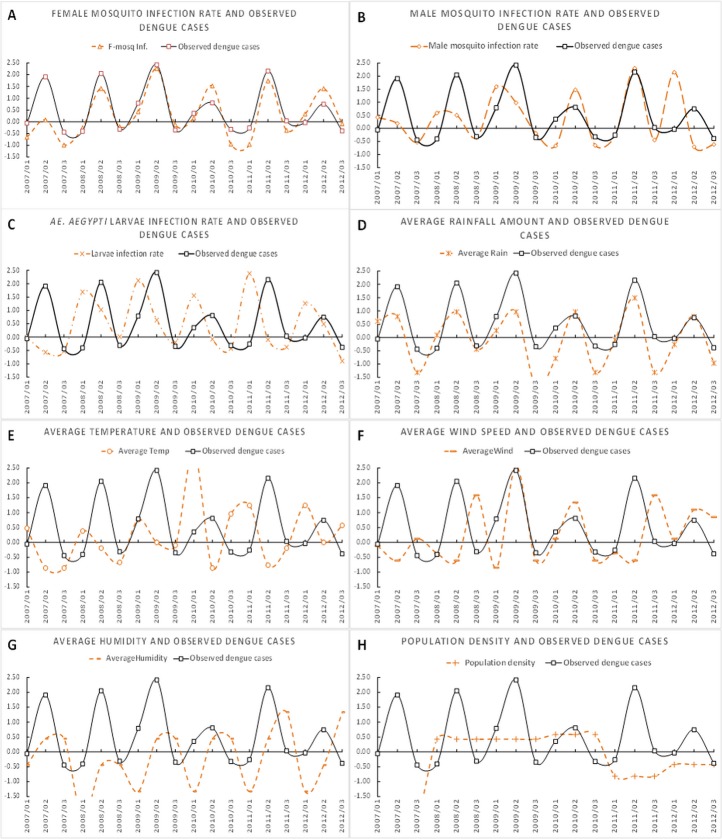
The trends of four predictors, dengue cases, and the infection rates of female, male, and larvae mosquitoes, are plotted and compared. This figure illustrates the trends of four parameters in Nakhon Pathom. The trends of these parameters in the other two provinces (Ratchaburi and Samut Sakhon) are similar but are not shown due to space limitations.

We hypothesized that the *Ae*. *aegypti* infection rate was associated with the morbidity rate of dengue outbreaks. Therefore, we initially studied the relationship between these parameters by plotting the line charts, as shown in Fig 2, compared to the plot of the observed dengue cases. The line chart in Fig 2 (A) and 2 (C) demonstrates that the trends of observed dengue cases and female mosquito infection rates fluctuated similarly. These trends show that they were positively correlated with the observed dengue cases. In contrast, the infected male mosquitoes and the dengue cases are divergent, and thus, they are potentially not associated with each other. The climate factors seemed to be less associated with the observed data, except for the rainfall amount (Fig 2D). More discussion about this can be found in the Discussion.

### Prediction performance evaluation

#### Data scaling performance study

Because different data attributes have different numeric ranges, greater numeric ranges can dominate those with smaller numeric ranges. Thus, scaling techniques are required to resolve this problem. Another benefit of scaling is that it avoids numerical difficulties during calculations. Because SVM kernel values usually depend on the inner products of feature vectors, e.g., the linear kernel (Eq 1) and the polynomial kernel (Eq 2), large attribute values might cause numerical problems [39] and result in high computational costs. Table 3 demonstrates the forecasting power of three SVM techniques, SVM-Linear (SVM-L), SVM-Polynomial (SVM-P), and SVM-RBF (SVM-R), and the computational time required to construct a model using unscaled and scaled data. SVM-L and SVM-P require 30.2201 and 595.5434 seconds, respectively, whereas SVM-R requires only 0.0135 seconds to construct the model. There is a dramatic decrease in the computational time of SVM-L and SVM-P when the data are scaled (0.0147 and 0.0183, respectively), and the prediction powers are also improved.

**Table 3:** The effect of scaled and unscaled data on prediction performance and model construction time.

Dataset	Kernels	Sensitivity	Specificity	Accuracy	Time to construct a model (seconds)
**Unscaled**	SVM-L	1	0.316	75.930	30.2201
	SVM-P	1	0.684	88.890	595.5434
	SVM-R	1	0.373	90.741	0.0135
**Scaled**	SVM-L	1	0.474	81.482	0.0147
	SVM-P	0.882	0.789	90.740	0.0183
	SVM-R	0.947	0.947	96.296	0.0119

Noticeably, the results from the testing in Table 3 showed that the highest prediction accuracy, sensitivity, and specificity were found in the RBF kernel with scaled data. Thus, SVM-R was chosen as the main model for morbidity rate prediction in this study.

#### SVM with RBF kernel parameter analysis

There are two parameters for the RBF kernel: *C* and *σ* ^2^, in which *C* is the parameter for regularization, determining the trade-off between the fitting error minimization and the smoothness of the estimated function. The second parameter, *σ* ^2^, is the kernel function parameter. There is no a structural way to select the optimal values of *C* and *σ* ^2^ for a given problem. This method is called a *grid search* and involves numerous trials and errors [38]. This section aims at investigating the effect of those two parameters on the performance of the RBF-based prediction model by varying one parameter at a time.

**Fig 3:**
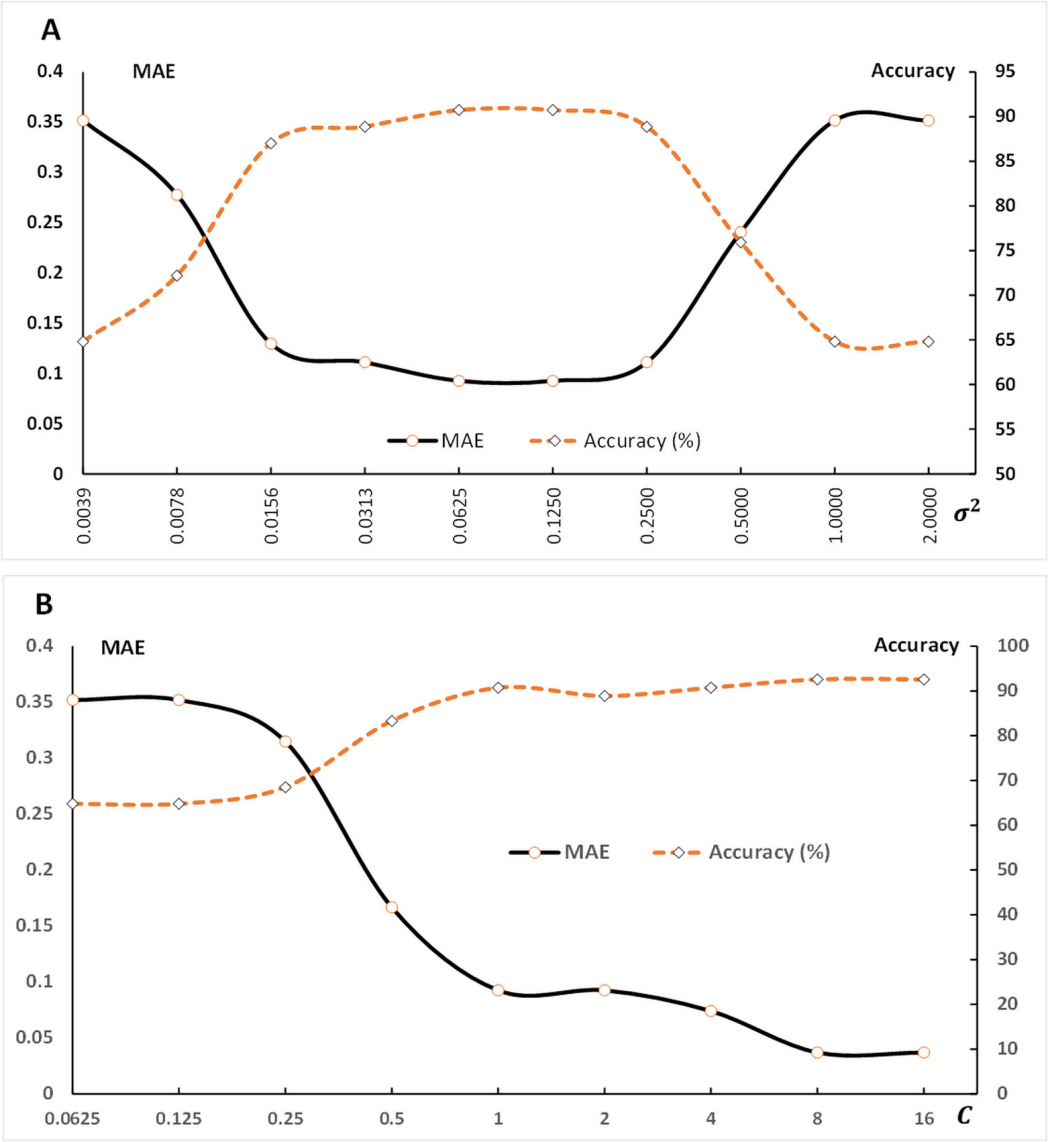
The prediction performance comparison based on the MAE and forecasting accuracy. Fig 3 (A) shows the MAE and the prediction accuracy with varying values of *σ* ^2^ while C was fixed. In contrast, the value of C was varied and *σ* ^2^ was fixed in Fig 3 (B) to determine the optimal values for the MAE and prediction accuracy.

Various pairs of (*C*, *σ* ^2^) values were tried, and the one with the best accuracy was selected. Fig 3 shows the evaluation parameter values at various values of *σ* ^2^ in which *C* is fixed at 1.0 (default value). Fig 3(A) illustrates the results for determining the optimal value of *σ* ^2^. The values of *σ* ^2^ were varied exponentially from 2^–8^, 2^–7^, 2^–6^,…, 2^1^ (the mean absolute error [MAE] and prediction accuracy remained steady afterwards). The figure shows that the lowest MAE value of 0.0926 and the highest prediction accuracy of 90.74% were obtained when *σ* ^2^ was 0.1250 (2^–3^). This value was selected as the optimal value of *σ* ^2^ and was used to determine the optimal value for *C*. Fig 3(B) shows the results of various values of *C* when *σ* ^2^ was chosen to be 0.1250 based on the previous experiment. The value of *C* was varied from 2^–5^ to 2^4^. A *C* value of 8 (2^3^), in which the MAE and the prediction accuracy remained steady afterwards, was chosen because it produced the highest predictive accuracy (96.296%) and the lowest MAE (0.037). Based on these results, the optimal SVM-RBF predictive model was defined by a value of C equal to 8 and a value of *σ* ^2^ equal to 0.1250 using 10-fold cross validation.

#### Significance of the female mosquito and *Ae*. *Aegypti* larvae infection rate features

This experiment aimed to study the significance of two new features found by the data analysis in Fig 2: the female mosquito and larvae infection rates. Two models were constructed to compare the predicted results. The first prediction model applied all nine attributes addressed in Table 1, while the second model eliminated the two new variables to study the effect of the two new parameters. Both models deployed three classification techniques, SVM-L, SVM-P, and SVM-R. The predicted performances were assessed by three measures (Eqs 4–6) and illustrated in Table 4. The candidate models were trained using the 2007–2013 data. To choose between those techniques, the model with the highest accuracy was determined. As shown in Table 4, all techniques in the first model yielded a higher prediction performance in terms of sensitivity, specificity, and accuracy than in the second model. This result suggests that the omitted infected female mosquito and infected larvae variables significantly affected the prediction performance. It was noted that the SVM-R had the highest prediction accuracy, up to 96.296%, among all the models. Therefore, this confirms the superior predictive power of the RBF kernel of SVM compared to other kernels using optimal parameters from the previous experiment of this study.

**Table 4:** Prediction performance comparisons of five techniques of two models.

Techniques	1^st^ Model			2^nd^ Model		
	Sensitivity	Specificity	Accuracy (%)	Sensitivity	Specificity	Accuracy (%)
**SVM-L**	0.579	1	85.185	0.474	1	81.482
**SVM-P**	0.737	1	90.74	0.684	0.929	87.037
**SVM-R**	***0*.*947***	***0*.*947***	***96*.*296***	***0*.*882***	***0*.*789***	***90*.*740***

#### Comparing the chosen model with classical forecasting techniques

**Fig 4:**
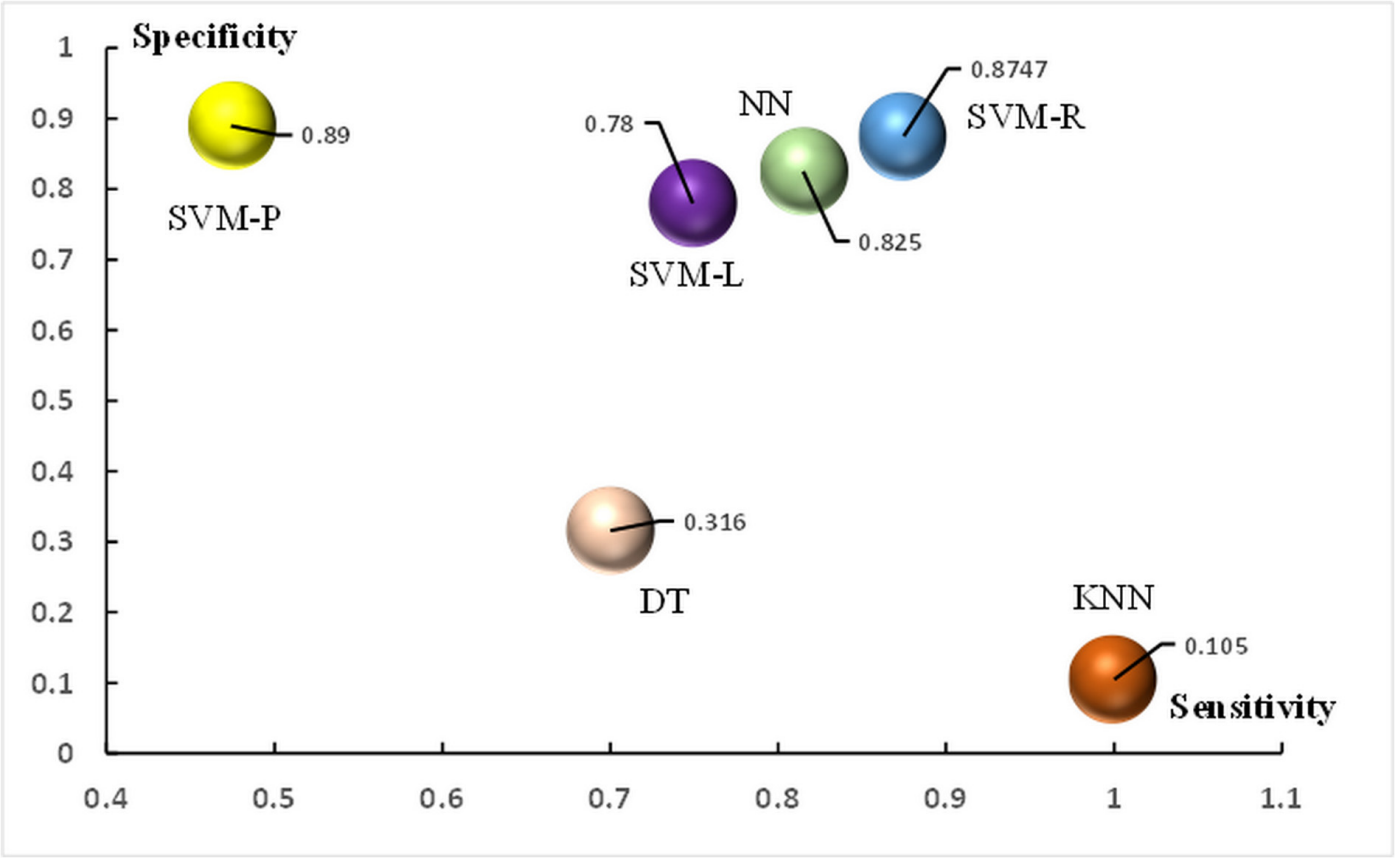
Prediction performance comparison based on specificity and sensitivity of the six models. A higher specificity and sensitivity suggest a better prediction efficiency. Among the evaluated models, the SVM-R obtained the highest prediction performance by achieving sensitivity and specificity of 0.8747 using a 10-fold cross-validation performed on the test set data.

**Fig 5:**
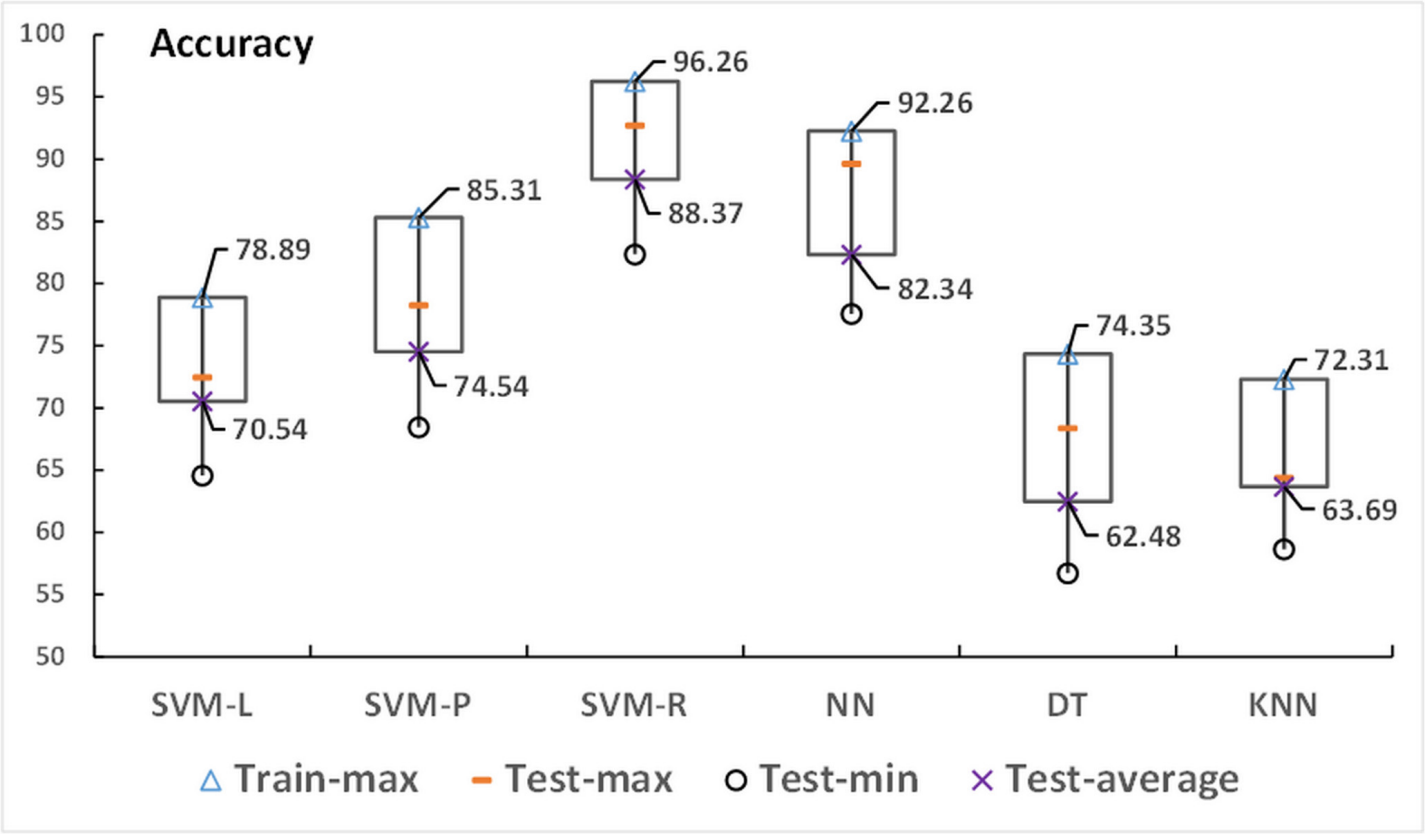
Prediction performance comparison based on the maximum, average, and minimum accuracy of the six models compared with the accuracy of the training set. The experiment was conducted 10 times using 10-fold cross-validation performed on the training set and the test set data using the SVM-L, SVM-P, SVM-R, NN, DT, and *KNN* techniques. All the collected results were averaged.

The purpose of the following experiment was to compare the SVM-R model with the other SVM kernels as well as other state-of-the-art classification techniques (Fig 4), such as the *KNN*, DT, and standard back propagation NN. These techniques are popular and have been applied in several research areas such as image processing, business data mining, and medicine. The collected data were completely separated (no overlapping instances) into a training set and a test set. To avoid model overfitting, a 10-fold cross validation was deployed. Fig 5 shows the values of sensitivity and specificity of all techniques, whereas Fig 5 demonstrates the minimum, average, and maximum values of prediction accuracy obtained from the test set of the six forecasting models as well as the average accuracy yielded from the training set (train-max). The square boxes in Fig 5 indicate the predictive accuracy differences between the training set and the test set. The SVM-R was superior to the other techniques because it obtained the highest sensitivity (0.8747) and specificity (0.8747), as shown in Fig 5, resulting in achieving a prediction accuracy of 96.26% in the training set and 88.37% in the test set.

## Discussion

The results of this study illustrated that the infection rates in female mosquitoes and larvae influenced the number of dengue cases in three provinces of Thailand. Fig 2(A) indicates that the female mosquito infection rate is strongly related to the number of observed dengue cases because they have similar line chart trends and because female mosquitoes typically forage and bite humans. Transovarially infected mosquitoes are known to be capable of transmitting the virus through biting [40]. Thus, a higher female mosquito infection rate results in higher biting activity rates, and, consequently, more dengue cases. In contrast to Fig 2(B), the dengue cases were not clearly associated with the male mosquito infection rate because their trends were divergent. Male mosquitoes do not hunt; therefore, the number of dengue cases may not be linked to the male mosquito infection rate. Fig 2(C) revealed that the high infection rate of *Ae*. *aegypti* larvae mosquitoes found in the summer was correlated to the high number of dengue cases in the following rainy season. When the infection rate of larvae declines, the morbidity rate is also lower. This phenomenon occurs repeatedly every year because a high infection rate of larvae in the summer leads to a high infection rate of female mosquitoes in the following rainy season. This result supports the report of Scott *et al*. [41] regarding the largest female populations being present in the rainy season (May-June) in Thailand. As a result, the biting activity rate for female mosquitoes increases and, consequently, more dengue cases occur. Thus, the larva infection rate in the summer was also positively correlated with the number of observed cases in the following rainy season. This discovery is very useful because it is a significant signal that indicates the severity of dengue outbreaks in the near future. In other words, if we know the infection rate of *Ae*. *aegypti* larvae in the summer, we can effectively predict the dengue incidence in the following rainy season. The amount of rainfall also seems to be associated with the number of cases, which results in a pattern that is similar to the pattern for the number of dengue cases, as shown in Fig 2 (D). The large volumes of standing water on private properties can revive mosquito eggs and subsequently create a surge in the emergence of infected mosquitoes. While some studies [5,12] have reported that climate variables play a key role in transmission during outbreaks, the data shown in Fig 2E–2G seem negatively associated with the observed number of dengue cases. This result occurs primarily because the studied areas are geographically similar, and thus, those climate factors are not very different. Therefore, the biting activity rates of the *Ae*. *aegypti* females in the studied areas are similar. As such, using only climate factors will lead to poorly predictive results.

Table 3 shows the importance of using the scaling method before forecasting data using various SVM kernels. The SVM-L and SVM-P suffered from high computational costs compared to the SVM-R because both SVM kernels depend on inner products of feature vectors (Eq 1 and 2). Therefore, these kernels are typically confronted with numerical problems due to the large difference of numeric ranges in the feature vectors. For example, the population size in each province in this study included over 100,000 people, while the mosquito infection rate was less than 100. In this case, the population density has a wide range of values, and the distance will be governed by this particular feature. Therefore, standardizing the range of all features allows for each feature to contribute in an approximately proportional manner to the final distance. The data in this study were standardized to the range of the independent variables or the features of the data. As a result, the computational time dramatically decreased, and more importantly, the prediction power significantly increased. Table 3 also shows that the prediction performance of the SVM-R increased from 90.74 to 96.29% after scaling the data, whereas the computational cost decreased slightly. Because computational time is crucial when dealing with large datasets, scaling the data is a very useful process for constructing a forecasting model for larger amounts of data in the future and for enhancing the prediction efficiency.

When the SVM is chosen as a forecasting technique for the dengue severity level prediction, we need to first consider what kernel function is the most suitable for the dengue data. A dynamic feature of the data used to predict the dengue severity level, e.g., climate factors and the mosquito infection rate, is that they are nonlinear. It has been suggested that using nonlinear kernel functions could achieve a better performance than linear kernel functions. Therefore, the SVM-R is used as the kernel function of SVM because it can effectively handle instances in which the relationship between the independent and dependent variables is nonlinear. In addition, the RBF kernel has less complexity (Table 3) than the polynomial kernel because it has less hyperparameters, which influences the complexity of model selection. This experiment demonstrates the performance of prediction (Table 3) obtained from the RBF kernel compared with the polynomial and linear kernels. However, Chih-Wei *et al*. [39] suggested that the RBF kernel will not improve the performance of classification when the number of features is very large; in that case, the linear kernel is preferred. Selecting the most suitable SVM-R kernel parameters for a predictive model is another challenging task of this research. The determinations of the optimal values of two important parameters, C and *σ* ^2^, for the SVM-R, are shown in Fig 3 (A) and 3 (B). This experiment explains that the performance of the kernel depends on the adjustable parameter *σ* ^2^ and should be carefully tuned to the problem at hand. Manually tuned parameters in this study seemed naïve and required a high experimental time. However, only two parameters did not need much more computational time than the heuristic methods. In addition, C and *σ* ^2^ are independent, and thus, the grid search can be easily parallelized, while this is difficult in several advanced methods because they are iterative processes [39].

In addition, we compared the SVM-R with other state-of-the-art techniques. The SVR-R provided the best values of all the evaluation criteria, sensitivity and specificity (Fig 4). The NN is an alternative model that can achieve a high prediction performance in terms of sensitivity and specificity. However, the SVM-R obtained a higher specificity and sensitivity than the NN. The SVM-R often outperforms the NN in practice because it addresses the biggest problem of the NN; SVMs are less prone to overfitting. The main drawback of the NN is that it has an expensive computational cost due to its algorithm complexity and curse of the dimension problem. Learning too much of the complexity of the NN results in overfitting the training set [38]. Another issue to note is that even if the NN solutions used tend to converge, this may not result in a unique solution [42]. Although the DT and *KNN* are simple and have a reduced computational cost, their prediction powers are significantly lower than the NN and SVM. The *KNN* had a specificity of 0.105, indicating that 10.5% of the high morbidity rate data instances were predicted as low morbidity rates, whereas the DT had only 31.6% specificity. These results are consistent with Fig 5, which shows that both the DT and *KNN* techniques obtained low prediction accuracies compared to the others. Among those techniques, the SVM-R outperformed the other state-of-the-art techniques measured by the maximum, average, and minimum accuracy. In addition, the difference of prediction accuracy obtained from the training set and test set of the SVM-R was small (demonstrated by the height of the rectangular boxes), which indicates that the model was not overfit.

The results in Table 4 suggest the significance of the two introduced predictors for the forecasting model. Infected dengue viruses in larvae and female mosquitoes have been demonstrated as the main determinants of outbreaks. They can increase the prediction accuracy by 4.32% on average for all models. The sensitivity and specificity also have been shown to vary depending on those two new parameters because they decrease when these predictors are omitted. Therefore, removing these parameters from the predictive model results in a decline of all the evaluation criteria, which has never been studied in any previous research.

Unlike other state-of-the-art frameworks, the originality and novelty of this work was introducing the use of the *Ae*. *aegypti* female mosquito and larva infection rates in the forecasting model to extrapolate the morbidity rate of DF. Our study results demonstrate that newly introduced predictors could effectively signal the risk of epidemics to local authorities in Thailand and help to reduce the severity of the outbreak in the country. It provides better prediction accuracy compared to state-of-the-art forecasting models such as the NN, DT, and *KNN* models In addition, the predictive model will inevitably complement and enhance the success of national dengue control. However, there are some limitations to our study. First, mosquito infection rate data have a limited availability in other areas. Even with the impressive efforts of the research team from Chulalongkorn University in collecting mosquito data from the studied areas, detecting the dengue virus in mosquitoes using the RT-PCR technique is time-consuming and an expensive laboratory cost. It was impossible to collect data and obtain the infection rate of mosquitoes for the whole country within a limited time (12 months) for this research project, which leads to the second limitation; the studied areas were small and fixed. However, the dynamic of the morbidity rate in the community is usually influenced by local factors. Therefore, the prediction model for a dengue outbreak is usually applicable only to a specific study area [5]. Although the prototype in this research exploits local dengue data, the proposed methodology in this research could be applicable for other geographical areas. Furthermore, the findings of this research could apply to other viral infectious diseases transmitted by mosquitoes, e.g., malaria or yellow fever.

Further studies are suggested to implement the forecasting model as a web-based application accessible by a user or non-technical user. All relevant data for prediction should be transformed into a data warehouse that is able to rapidly generate reports for users, which will also create advantages for supporting decision making by policy makers to strengthen dengue control and take precautions to prevent dengue epidemics from becoming pervasive.
